# Case report: Diagnostic clues for anti-3-hydroxy-3-methylglutaryl-coenzyme A reductase myopathy in pediatric patients

**DOI:** 10.3389/fped.2023.1102539

**Published:** 2023-03-13

**Authors:** Keisuke Watanuki, Hiroshi Koga

**Affiliations:** Department of Pediatrics, National Hospital Organization Beppu Medical Center, Beppu, Japan

**Keywords:** autoimmune disease, creatine kinase, hydroxymethylglutaryl coA reductase, muscular disease, myositis

## Abstract

**Introduction:**

Anti-3-hydroxy-3-methylglutaryl-coenzyme A reductase (HMGCR) myopathy is a recently recognized pathology, but appears less common in children and the characteristics of pediatric cases remain unclear.

**Case report:**

We report a pediatric case of anti-HMGCR myopathy accompanied by skin rash. Motor function and serum creatine kinase level normalized after combinational treatment including early intravenous immunoglobulin, methotrexate, and corticosteroid.

**Literature review:**

We searched PubMed and identified reports with detailed clinical information of 33 pediatric patients <18 years old with anti-HMGCR myopathy. Among these 33 patients and our own case, skin rash and maximum serum creatine kinase level >5,000 IU/L were observed in 44% (15 patients) and 94% (32 patients), respectively. Skin rash was present in 15 of the 22 patients (68%) ≥7 years old and none of the 12 patients (0%) <7 years old. Among the 15 patients with skin rash, 12 (80%) presented with erythematous rash.

**Conclusion:**

Erythematous skin rash may offer a clue to the diagnosis of anti-HMGCR myopathy in children with muscle weakness and serum creatine kinase level >5,000 IU/L in the absence of other myositis-specific antibodies, particularly in patients ≥7 years old. Our results suggest the importance of early anti-HMGCR testing in pediatric patients with these manifestations.

## 1. Introduction

Anti-3-hydroxy-3-methylglutaryl-coenzyme A reductase (HMGCR) is a myositis-specific antibody associated with immune-mediated necrotizing myopathy (IMNM), a new subgroup of idiopathic inflammatory myopathy (1). The prevalence of idiopathic inflammatory myopathies ranges from 2.4–33.8 per 100,000, with anti-HMGCR myopathy accounting for 6%–10%, but <1% in children (1). IMNM is characterized by acute or subacute proximal-predominant muscle weakness with creatine kinase (CK) elevation and myopathological findings of muscle fiber necrosis and regeneration with minimal or no inflammation (1). Statin exposure can provide a trigger for anti-HMGCR myopathy among adults (1). Combinational treatment including intravenous immunoglobulin (IVIg), corticosteroid, rituximab, and other immunosuppressants such as methotrexate has been recommended for IMNMs (2) and a recent study supported the effectiveness of early IVIg treatment for adult patients with IMNM (3). However, the characteristics of and optimal treatment strategy for children with IMNM remain to be elucidated because of the limited clinical data. We report herein the case of a boy with anti-HMGCR myopathy and review the literature for pediatric patients with anti-HMGCR myopathy to explore the clinical characteristics and identify clues to better management.

## 2. Case description

### 2.1. Patient information

The patient was a 13-year-old Japanese boy, the first of three siblings from non-consanguineous parents. He had no history of statin exposure and no family history of neuromuscular disease. He was a basketball player and presented with progressive difficulties in shooting, running, and subsequently walking during the previous one month.

### 2.2. Physical examination

**Figure 1:**
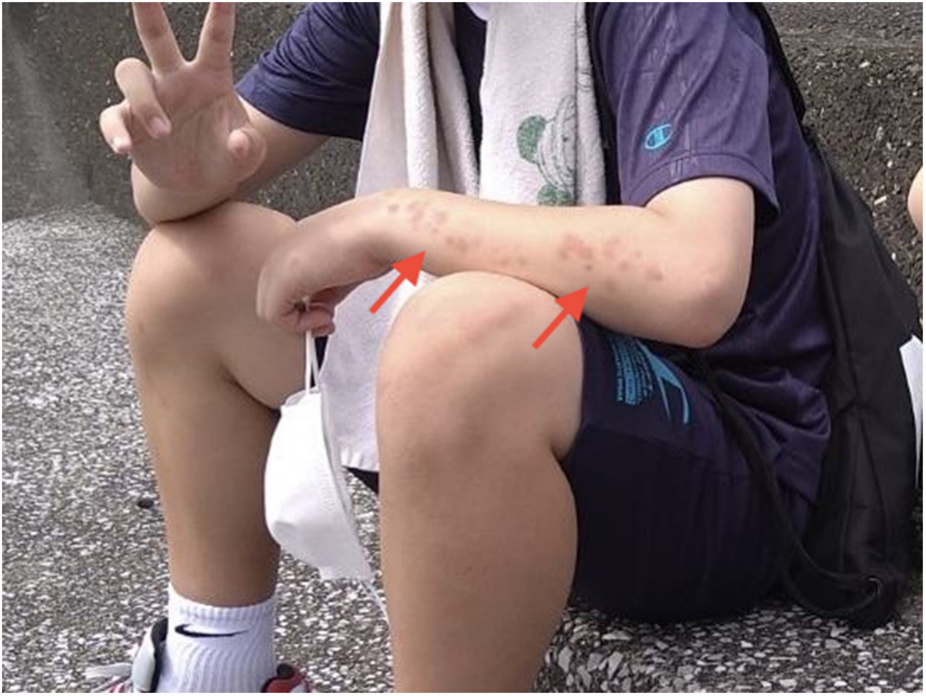
Erythematous skin rash observed on the left forearm of our patient at onset (arrows). Skin rash then spread to the other extremities and trunk.

Vital signs were normal (heart rate, 82 beats/min; respiratory rate, 15 breaths/min; blood pressure, 113/57 mmHg; body temperature, 36.5°C). Physical examination revealed grade 3/5 muscle weakness in the upper extremities and 4/5 in the lower extremities based on the Medical Research Council (MRC) scale (0–5) (4). The patient also presented with generalized erythematous skin rash (Figure 1). Other general physical examination findings were unremarkable.

### 2.3. Diagnostic assessment

**Figure 2:**
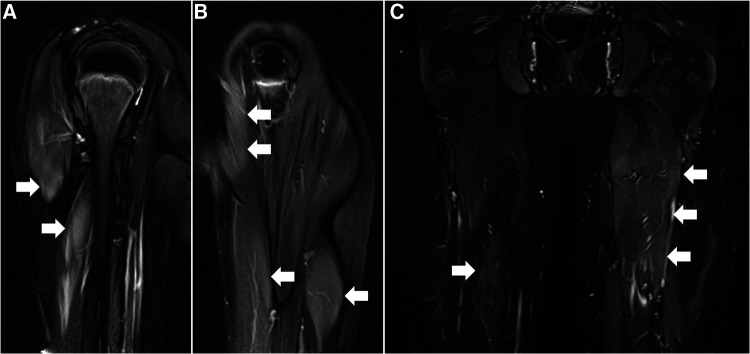
Muscle magnetic resonance imaging before treatment. (**A,B**) T2-weighted images of the right (**A**) and left (**B**) upper extremities in coronal section. (**C**) Short-tau inversion recovery image of lower extremities in coronal section. Arrows denote increased signal intensities of proximal muscles in bilateral upper and lower extremities.

**Figure 3:**
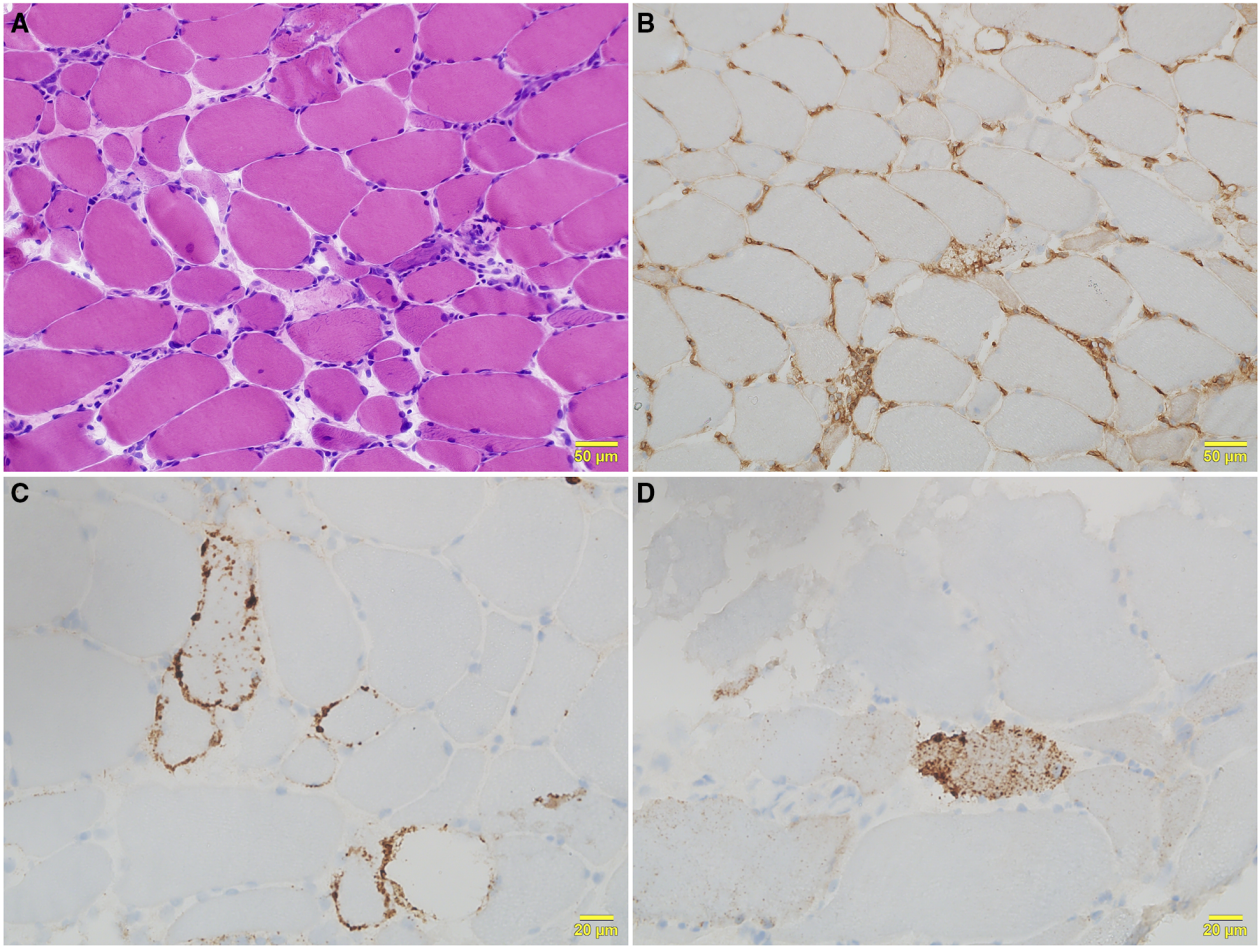
Histological features of right biceps brachii biopsy. (**A**) Hematoxylin and eosin staining shows moderate variation in fiber size, with scattered necrotic and regenerating fibers and mild lymphocytic infiltration. (**B**) Major histocompatibility complex class 1 immunohistochemical staining shows positivity on the sarcolemma of all fibers. (**C**) Sarcolemmal deposition of membrane attack complex (C5b−9) is seen in scattered fibers with C5b−9 immunohistochemical staining. (**D**) Fine granular expression of p62 is present in the sarcoplasm of scattered myofibers with p62 immunohistochemical staining.

Serum levels of CK (19,306 U/L), aldolase (257 U/L), aspartate aminotransferase (274 U/L), and lactate dehydrogenase (1,471 U/L) were all elevated. Magnetic resonance imaging detected signal hyperintensity on T2-weighted imaging and short tau inversion recovery imaging for muscles in the proximal upper and lower extremities (Figure 2). Muscle biopsy showed necrosis and regeneration of muscle fibers (Figure 3). Immunohistochemistry demonstrated overexpression of major histocompatibility complex class 1 and membrane attack complex (C5b−9) on the sarcolemma and granular sarcoplasmic expression of p62 (Figure 3). As measured by quantitative enzyme-linked immunosorbent assay (Cosmic Corporation, Tokyo, Japan), anti-HMGCR antibody level was 2.9 IU/ml (reference value <1.0 IU/ml), while negative results were obtained for anti-signal recognition particle antibody and other myositis-associated antibodies, including anti-Jo-1, anti-Mi-2, anti-MDA-5, and anti-TIF-1 gamma antibodies. The patient was subsequently diagnosed with anti-HMGCR myopathy.

### 2.4. Treatment and follow-up outcomes

Based on the consensus statement on the initial treatment for anti-HMGCR myopathy from the 224th European Neuromuscular Centre International Workshop (2), intravenous methylprednisolone (1 g/day for 3 days) followed by oral prednisolone (1 mg/kg/day), monthly IVIg (2 g/kg/dose, three times), and oral methotrexate (0.3 mg/kg/week) was started 3 months after the first evaluation. MRC scale scores for the upper and lower extremities normalized (grade 5/5) and serum CK (201 U/L), aldolase (8 U/L), aspartate aminotransferase (16 U/L), and lactate dehydrogenase (280 U/L) levels were all decreased by 3 months after treatment initiation. The patient resumed playing basketball at the same level as that before the onset of anti-HMGCR myopathy. The patient has continued to receive methotrexate monotherapy and as of the time of writing, has remained relapse-free for 2 years.

### 2.5. Literature review

We searched PubMed using the terms “myositis[mh]” and “necrosis[mh]” or “anti-HMGCR[tiab]” up to July 31, 2022. This electronic search identified 710 records. Inclusion criteria were as follows: (1) studies including patients with anti-HMGCR myopathy <18 years old; and (2) anti-HMGCR myopathy diagnosed by both anti-HMGCR antibody and compatible myopathological features such as prominent necrosis and regeneration of muscle fibers with mild or absent inflammatory infiltrates. Duplicated publications, conference abstracts, and other studies that did not report the detailed clinical features of patients were excluded. We excluded 697 articles based on these eligibility criteria. A total of 13 articles were included in the final review, containing 33 pediatric cases with anti-HMGCR myopathy as confirmed by positive results for anti-HMGCR antibody and consistent myopathological findings with detailed information (Table 1). Clinical response to treatment was evaluated based on the definition of complete remission as normalized motor function and serum CK level and partial remission as improved but not normalized motor function and serum CK level. For the 34 pediatric patients, including our own case, median age at onset was 9 years [interquartile rage (IQR), 5–11 years] and a maximum serum CK level >5,000 IU/L was observed in 32 patients (94%). Skin rash was noted in 15 patients (44%). Among the 15 patients with skin rash, 12 (80%) presented with erythematous rash, 1 with hyperpigmentation, 1 with linear morphea scleroderma, and 1 with rash of unknown detail. Skin rash was localized in 10 patients (67%; on the extremities in 6 [40%] on the face or neck in 4 [27%]), generalized in 2 (13%), and unknown in the other 3. A significant positive correlation was confirmed between age at onset and maximum serum CK level (Pearson correlation coefficient = 0.45, *P* = 0.0074). In one patient diagnosed in infancy, maximum serum CK level was only 918 IU/L (Table 1) (5). Patients were stratified by age at onset before and after school age (7–17 years vs. <7 years) and clinical characteristics were compared between groups (Table 2). The cumulative incidence of skin rash and median maximum serum CK level was significantly higher in pediatric patients ≥7 years old at onset than in those <7 years old.

**Table 1:** The 34 pediatric patients <18 years old with anti-HMGCR myopathy identified by the literature review.

Patient	Age at onset (years)	Sex	Muscle weakness	Skin rash	Maximum CK level (IU/L)	Treatment				Treatment response	References
						IVIg	Corticosteroid	MTX	Rituximab		
1	4	F	+	−	12,180	−	+	+	−	PR	(6)
2	9	F	+	+	12,662	+	+	+	−	PR	(6)
3	11	M	+	−	19,000	−	−	+	+	PR	(6)
4	13	F	+	+	44,002	−	+	+	+	PR	(6)
5	9	M	+	+	8,000	+	−	−	−	PR	(7)
6	0.8	NA	+	−	918	−	+	−	−	CR	(5)
7	3	NA	+	−	6,175	−	+	−	−	PR	(5)
8	5	NA	+	−	5,453	−	+	−	−	PR	(5)
9	6	NA	−	−	8,240	+	+	+	−	PR	(5)
10	7	NA	+	−	5,460	−	+	−	−	PR	(5)
11	9	NA	+	−	9,570	+	+	+	−	PR	(5)
12	10	NA	+	+	10,891	+	+	+	−	CR	(5)
13	11	NA	+	+	7,508	+	+	+	−	CR	(5)
14	13	NA	+	−	7,183	−	+	+	−	PR	(5)
15	5	F	+	−	7,500	+	−	−	−	PR	(8)
16	6	M	+	−	8,687	−	−	−	−	Unchanged	(9)
17	9	F	+	+	9,570	+	+	+	+	PR	(9)
18	11	F	+	+	9,786	+	+	+	+	PR	(9)
19	14	F	+	−	9,383	−	+	−	−	CR	(9)
20	8	F	+	−	23,000	+	−	−	−	PR	(10)
21	10	M	+	+	9,000	+	−	−	−	PR	(10)
22	4.5	M	+	−	8,452	+	+	+	−	Unchanged	(11)
23	4.5	F	+	−	5,858	−	+	+	+	PR	(12)
24	5	F	+	−	6,175	+	+	+	+	PR	(12)
25	3.5	F	+	−	7,842	+	−	−	−	PR	(13)
26	7	M	+	+	10,000	+	+	+	−	PR	(14)
27	9	F	+	+	18,000	+	+	+	+	PR	(15)
28	4	F	+	−	13,470	+	+	+	−	PR	(16)
29	7	F	+	+	20,000	+	+	+	−	PR	(16)
30	12	F	+	+	7,191	−	+	−	−	PR	(16)
31	13	F	+	+	4,231	+	+	+	−	PR	(16)
32	15	F	+	−	38,966	+	+	+	−	CR	(16)
33	9	M	+	+	30,833	+	+	+	−	PR	(17)
34	13	M	+	+	19,306	+	+	+	−	CR	Present case

Positive anti-HMGCR antibody and compatible myopathological findings were confirmed in all 34 patients.

HMGCR, 3-hydroxy-3-methylglutaryl-coenzyme A reductase; CK, creatine kinase; CR, complete remission; F, female; IVIg, intravenous immunoglobulin; M, male; MTX, methotrexate; NA, not available; PR, partial remission.

**Table 2:** Comparison of clinical characteristics according to age at onset among 34 pediatrics patients with anti-HMGCR myopathy.

	Age at onset 7–17 years (*n* = 22)	Age at onset <7 years (*n* = 12)	Relative risk (95% confidence interval)	*P* value
Skin rash, *n* (%)	15 (68)	0 (0)	Not applicable	0.0001
Maximum serum CK level (IU/L), median (IQR)	9,893 (7,877–19,480)	7,709 (5,937–8,628)		0.012
Treatment, *n* (%)				
Corticosteroid	18 (82)	9 (75)	1.1 (0.7–1.6)	0.68
Intravenous immunoglobulin	16 (73)	6 (50)	1.5 (0.8–2.7)	0.27
Methotrexate	16 (73)	6 (50)	1.5 (0.8–2.7)	0.27
Rituximab	5 (23)	2 (17)	1.4 (0.3–6.0)	1.0
Clinical improvement, *n* (%)				
Complete remission	5 (23)	1 (8.3)	2.7 (0.4–20.7)	0.39
Partial remission	17 (77)	9 (75)	1.0 (0.7–1.5)	1.0

Comparisons between groups were made using Fisher's exact test for categorical variables and the Wilcoxon rank-sum test for continuous variables.

HMGCR, 3-hydroxy-3-methylglutaryl-coenzyme A reductase; CK, creatine kinase; IQR, interquartile range.

## 3. Discussion

Our findings suggest that early IVIg is also effective for children with anti-HMGCR myopathy. Although skin rash has been reported in pediatric patients with anti-HMGCR myopathy (5, 6), the prevalence remains unknown. Our review of the literature revealed skin rash as an extramuscular feature among 68% of pediatric patients 7–17 years old with anti-HMGCR myopathy.

Skin rash has not been recognized as an extramuscular clinical feature among adult patients with anti-HMGCR myopathy (1). The etiology of skin rash in pediatric patients with anti-HMGCR myopathy remains unclear. Considering the high incidence of skin rash in pediatric patients 7–17 years old, skin lesions may be age-dependent and associated with enhanced immunoreactivity. Lean muscle mass increases with age due to hormonal effects (18), so the positive correlation between maximum serum CK level and age at onset may be due to increased skeletal muscle mass as age increases, leading to enhanced immunoreactivity, greater muscular injury, and skin lesions.

A growing number of reports have described anti-HMGCR myopathy initially diagnosed as limb-girdle muscular dystrophy, dermatomyositis, or congenital muscular disease (5, 10, 13, 19). In previous studies, delayed diagnosis of anti-HMGCR myopathy or initial misdiagnosis as limb-girdle muscular dystrophy has been reported due to the similarity of clinical features (10, 13), such as proximal muscular weakness and increased serum CK levels. Anti-HMGCR antibody testing, highly elevated serum CK levels, and potentially skin rash are important to differentiate anti-HMGCR myopathy from limb-girdle muscular dystrophy in pediatric patients without myositis-specific antibodies, while the presence of a family history is indicative of limb-girdle muscular dystrophy (10). Anti-HMGCR myopathy with skin involvement, especially in patients ≥7 years old, may mimic juvenile dermatomyositis (19) and infantile-onset anti-HMGCR myopathy may mimic congenital muscular diseases (5). Positive anti-HMGCR antibody testing is also useful to distinguish anti-HMGCR myopathy from dermatomyositis and congenital muscular diseases. Myopathological findings of perifascicular atrophy and positive findings for myositis-specific anti-Mi-2, anti-MDA-5, anti-TIF-1 gamma, or anti-NXP-2 antibodies are characteristic of dermatomyositis (20). Congenital muscular diseases, such as congenital myopathy and congenital muscular dystrophy, usually present as muscular weakness at birth or during infancy and can be diagnosed by genetic analysis and myopathological examination. Among the children with anti-HMGCR myopathy included in our review, infantile onset was observed in only one patient, in whom the age at onset was 10 months (5).

In our literature review, the complete response rate was 18% with unstandardized combinational treatments among pediatric patients with anti-HMGCR myopathy. No difference in treatment response was seen between those ≥7 years old at onset and <7 years old. Further study is needed to elucidate overall and age-stratified response rates to the treatment recently recommended for patients with anti-HMGCR myopathy (2).

Our findings have three important clinical implications. First, early diagnosis and treatment of anti-HMGCR myopathy within 6 months after onset may help avoid progression of muscular injury. Second, anti-HMGCR antibody testing is indicated for pediatric patients presenting with muscle weakness, a serum CK level elevated to >5,000 IU/L, and skin rash, particularly for those ≥7 years old. Third, however, a high serum CK level and skin rash may be absent in young children with anti-HMGCR myopathy. The design as a literature review represents an important limitation to this study in providing an estimate of the prevalence of skin rash among children with anti-HMGCR myopathy. Further large-scale studies would overcome this limitation and are needed to validate an early diagnostic approach and to elucidate the optimal treatment strategy for anti-HMGCR myopathy even if early diagnosis becomes possible.

In conclusion, erythematous skin rash may provide a clue to the diagnosis of anti-HMGCR myopathy in children with muscle weakness and serum creatine kinase level >5,000 IU/L, particularly in those ≥7 years old. Early diagnosis of anti-HMGCR myopathy in children may lead to better clinical management with early IVIg treatment.

## Data Availability

The raw data supporting the conclusions of this article will be made available by the authors, without undue reservation.
